# Successful ECMO support for cardiogenic shock induced by immune checkpoint inhibitor-associated myocarditis: a case report and literature review

**DOI:** 10.3389/fimmu.2025.1646040

**Published:** 2025-12-08

**Authors:** Yujing Zhang, Ruiting Li, Yongxiang Jiang, Shengwen Sun, Yin Yuan, You Shang, Huaqing Shu

**Affiliations:** Department of Critical Care Medicine, Union Hospital, Tongji Medical College, Huazhong University of Science and Technology, Wuhan, China

**Keywords:** immune checkpoint inhibitor, immune-related adverse event, immune checkpoint inhibitor-associated myocarditis, cardiogenic shock, ECMO

## Abstract

**Objectives:**

Immune checkpoint inhibitor (ICI)-associated myocarditis is a rare but potentially fatal immune-related adverse event that can rapidly progress to life-threatening arrhythmias and cardiogenic shock, often necessitating mechanical circulatory support. Extracorporeal membrane oxygenation (ECMO) has emerged as a critical life-saving intervention in such cases. However, the role of ECMO in treating ICI-associated myocarditis remains underexplored, with limited literature available.

**Methods:**

We present a case of fulminant ICI-associated myocarditis with cardiogenic shock successfully managed with ECMO. Additionally, we review and summarize data from 13 ECMO-assisted patients with ICI-associated myocarditis to provide insights into the clinical characteristics, management strategies, and outcomes.

**Main results:**

Among the 13 patients (with a mean age 59.08 years), monotherapy using nivolumab or pembrolizumab represented the predominant ICI treatment regimen. The median treatment cycle was 3.0 (IQR: 2.0 ~ 7.0), and the median duration from first administration to myocarditis onset was 77.0 (IQR: 20.5 ~ 250.0) days. The median duration of myocarditis symptoms was 19.0 (IQR: 15.5 ~ 42.5) days. Common presenting symptoms included fever and dyspnea, while most patients exhibited elevated myocardial enzymes and BNP levels, arrhythmias, and an average left ventricular ejection fraction (LVEF) of 38.31% at admission. Myocardial biopsy was the primary diagnostic method. In addition to immunosuppressive therapy, most patients also required intra-aortic balloon pump (IABP) support. The median duration of ECMO and IABP support was 9.0 (IQR: 6.5 ~ 15.5) days and 11.5 (IQR: 7.5 ~ 13.0) days, respectively. Ultimately, nine of the thirteen patients (69.23%) survived.

**Conclusions:**

Our analysis demonstrates ECMO’s potential as a bridge-to-recovery strategy for severe ICI-associated myocarditis with cardiogenic shock. The observed survival rate of 69.23% supports its judicious use in conjunction with prompt immunosuppression. Prospective studies are warranted to optimize ECMO initiation criteria, duration, and combination strategies with other circulatory support modalities.

## Introduction

Immune checkpoint inhibitors (ICIs) have been increasingly utilized in cancer patients, and it has demonstrated that ICIs have dramatically improved the prognosis of many kinds of cancer patients, such as melanoma, renal cell carcinoma, non-small cell lung cancer, bladder cancers, Hodgkin lymphoma, and others (1). However, ICIs are associated with a broad spectrum of organ toxicities known as immune-related adverse events (irAEs), resulting from excessive immune activation (2). Unlike adverse effects from chemotherapy and targeted therapies, irAEs can affect nearly every organ system, manifesting as dermatitis, arthritis, colitis, hepatitis, myocarditis, pneumonitis, encephalitis, and others (3–5). Notably, although myocarditis is a rare complication, its high mortality rate makes it clinically significant. Study has reported an overall incidence of ICI-associated myocarditis at approximately 1%, yet it carries a fatality rate as high as 40-50% due to rapid progression to life-threatening arrhythmias and cardiogenic shock (6).

Despite intensive pharmacologic interventions, a subset of patients with ICI-associated myocarditis progress to refractory cardiogenic shock, necessitating advanced therapeutic strategies. In such cases, mechanical circulatory support (MCS), particularly extracorporeal membrane oxygenation (ECMO), has emerged as a critical life-saving intervention. ECMO provides temporary circulatory and respiratory support, allowing time for immunosuppressive therapy to take effect (7–10). However, the role of ECMO in managing ICI-associated myocarditis remains underexplored, and there is limited literature describing its efficacy in this setting. In this report, we present a case of fulminant ICI-associated myocarditis leading to cardiogenic shock that was successfully treated with ECMO support. Additionally, we review previously reported cases to provide insights into the clinical characteristics, management strategies, and outcomes of ECMO-assisted patients with ICI-associated myocarditis. Our findings aim to enhance understanding of this life-threatening condition and optimize treatment strategies.

## Presentation of case

A 41-year-old female patient presented to our outpatient clinic on June 20, 2024, experiencing a sudden cardiac arrest at 10:30 AM. Cardiopulmonary resuscitation (CPR), endotracheal intubation, and defibrillation were immediately initiated. Return of spontaneous circulation (ROSC) was achieved at 11:47 AM, after which she was transferred to the intensive care unit (ICU) in a comatose state. Her medical history included a diagnosis of anal squamous cell carcinoma in December 2023, staged as T2N0MX. She had completed six cycles of chemoradiotherapy and three cycles of pucotenlimab treatment (200 mg every 3 weeks). The patient was hospitalized due to markedly elevated high-sensitivity troponin I (hs-TnI, 1153 ng/L, normal < 26.2 ng/L) during a follow-up visit on May 24, 2024. She was administered with methylprednisolone (mPSL, 60–80 mg/day). Despite her troponin levels continuing to rise and peaking of hs-TnI at 2767.7 ng/L, her cardiac function remained stable throughout her hospital stay. Consequently, she was discharged from the hospital on June 10, 2024. However, she experienced symptoms of chest tightness, lower limb pain, and generalized fatigue on June 13, but did not seek medical intervention.

**Figure 1:**
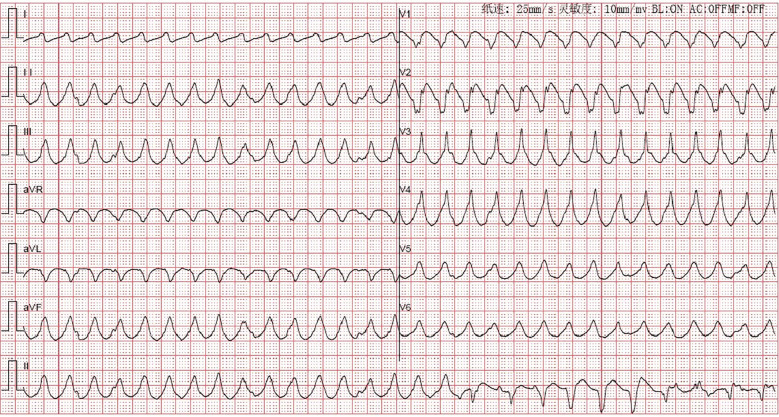
The electrocardiogram (ECG) of the patient after ICU admission.

Upon ICU admission, she remained a deep coma with bilateral non-reactive pupils (3.5 mm left, 4.0 mm right). She was mechanically ventilated in Synchronized Intermittent Mandatory Ventilation mode (SIMV; FiO_2_: 45%, Tidal volume: 400 mL, Respiratory rate: 15 breaths per minute and PEEP: 10 cmH_2_O). Hemodynamic instability persisted, characterized by recurrent episodes of ventricular tachyarrhythmias (**Figure 1**), ventricular fibrillation and cardiogenic shock requiring multiple defibrillations, intravenous administration of epinephrine and lidocaine, and high-dose infusions of norepinephrine. Arterial blood gas analysis revealed severe metabolic acidosis, with normal blood potassium (pH 7.108, lactate 12.7 mmol/L, potassium 4.9 mmol/L). Point-of-care ultrasound (POCUS) revealed a marked impairment of systolic function and a substantial decrease in left ventricular ejection fraction (EF) (**Video 1**). No evidence of deep vein thrombosis was identified.

Based on the recent ICI therapy and clinical presentation, a multidisciplinary team diagnosed ICI-associated myocarditis. Due to conventional treatment failed to stabilize her hemodynamic status, Veno-Arterial ECMO (VA-ECMO) was established via peripheral cannulation at 14:30 on June 20, 2024. A 18-French arterial cannula was inserted into the right femoral artery, and a 21-French venous cannula was positioned in the right femoral vein, both percutaneously under ultrasound guidance. Systemic anticoagulation was initiated with unfractionated heparin, targeting an activated partial thromboplastin time (aPTT) of 50–60 seconds. To prevent and monitor for lower limb ischemia, a distal perfusion catheter (14G Single-Lumen Catheter, Yixinda) was placed in the superficial femoral artery of the cannulated limb. Distal limb perfusion was continuously assessed via daily clinical examination (color, temperature, capillary refill) and continuous pulse oximetry on the great toe. Subsequent blood gas analysis demonstrated significant improvement (pH 7.428, PaCO_2_ 35.9 mmHg, PaO_2–_171 mmHg, lactate 6.9 mmol/L). The patient subsequently received intravenous high-dose mPSL pulse therapy (1,000 mg/day for three consecutive days), and five sessions of plasma exchange. The steroid taper regimen comprised: mPSL 250 mg every 12 hours for 3 days, then 100 mg every 12 hours, eventually transitioning to oral prednisone 20 mg/day with tapering every 5 days. Despite ongoing VA-ECMO support, the patient experienced refractory ventricular tachycardia, recurrent ventricular fibrillation and persistent hypotension, necessitating continuous administration of norepinephrine, epinephrine, amiodarone, and lidocaine. By 20:00 on June 20, the patient’s hemodynamic status further deteriorated manifesting as a markedly decreased pulse pressure (<10 mmHg), inadequate aortic valve opening, and left ventricular dilation, as demonstrated by POCUS (**Video 2**), prompting emergent insertion of an intra-aortic balloon pump (IABP) for additional circulatory support.

**Figure 2:**
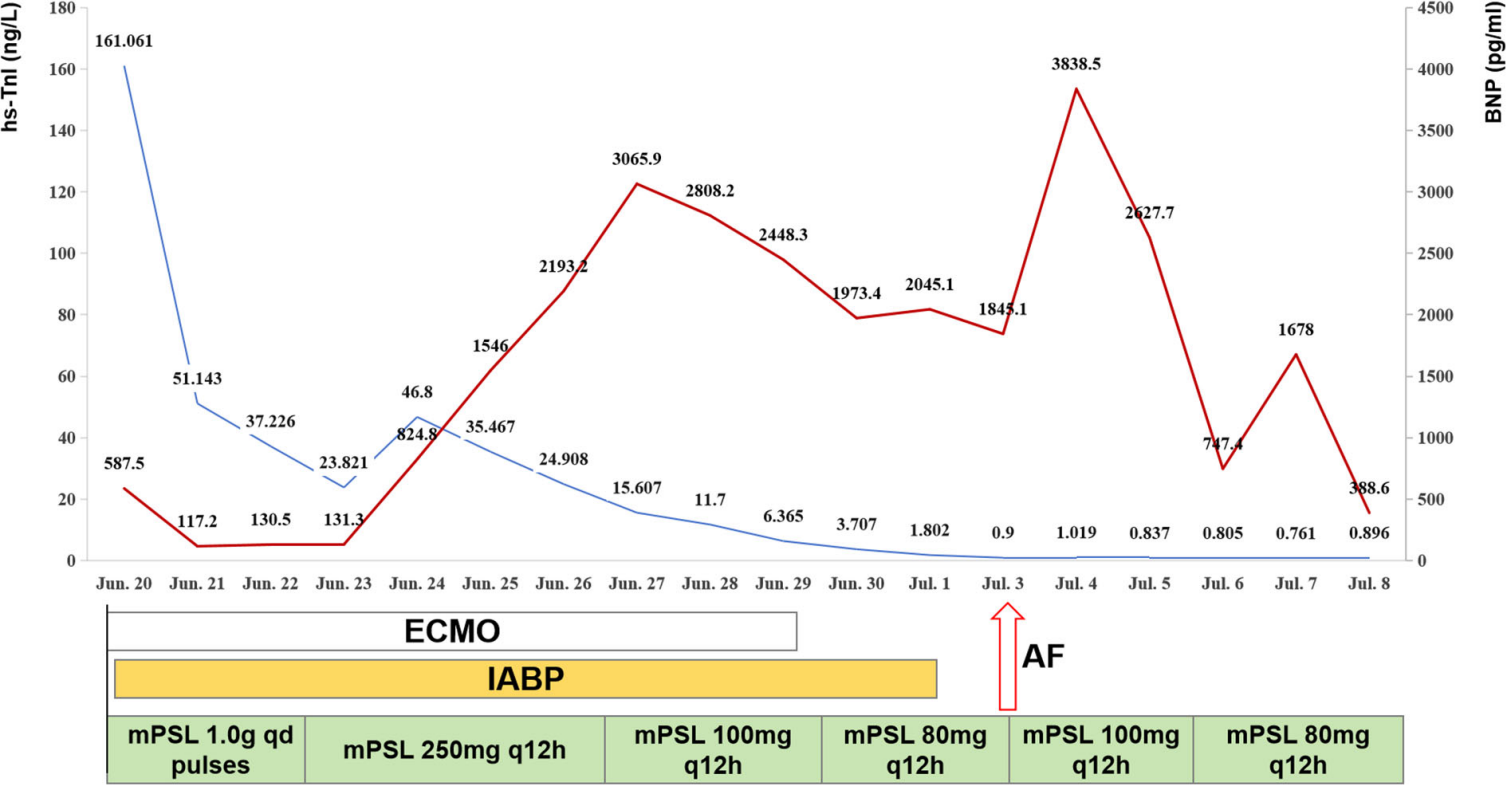
Changes in hs-TnI and BNP levels of the patient after admission. The solid blue line represents hs-TnI (left Y-axis), and the solid red line represents BNP (right Y-axis). White Rectangle: The start and end points of this rectangle indicate the initiation and weaning of VA-ECMO, respectively, with its length representing the total duration of ECMO support. Orange Rectangle: The start and end points of this rectangle indicate the initiation and removal of the IABP, respectively, with its length representing the total duration of IABP support. Green Hatched Rectangle represents mPSL tapering schedule. Red hollow Arrow: This arrow marks the timing of the recurrent VF event. hs-TnI, high-sensitivity troponin I; BNP, B-type natriuretic peptide; ECMO, extracorporeal membrane oxygenation; IABP, intra-aortic balloon pump; mPSL, methylprednisolone; VF, ventricular fibrillation.

Since the patient initially presented with respiratory failure secondary to pulmonary edema, a fluid negative balance strategy was employed for management. Persistently elevated B-type natriuretic peptide (BNP) levels and arrhythmias posed challenges to ventilator weaning. On June 26, the patient was awake and alert with clear consciousness during the daily morning assessment. To optimize respiratory care and minimize sedative drugs use for enhanced neurological monitoring, temporary tracheostomy was performed on June 27, enabling progressive weaning from mechanical ventilation. Concurrently, the patient’s cardiac function had improved and circulatory status had stabilized, requiring only a low dose of dobutamine (2 μg/kg/min) for maintenance. hs-TnI and BNP levels showed marked reduction (**Figure 2**), and POCUS revealed enhanced cardiac function (EF 40%, LVOT VTI 14 ~18 cm, pulse pressure 50 ~60 mmHg, MAP >65 mmHg) (**Video 3**). VA-ECMO was successfully weaned off on June 29, and the IABP was removed on July 1.

However, the patient experienced sudden ventricular fibrillation on July 3 at 10:26 AM, immediate CPR and two instances of asynchronous 200J defibrillation were administered, after which sinus rhythm was restored. Later that day at 3:27 PM, the patient developed recurrent ventricular fibrillation, necessitating CPR and one instance of asynchronous 200J defibrillation, again restoring sinus rhythm. A comprehensive review of the clinical course revealed the patient maintained a slightly negative fluid balance during this period and no recent signs of volume overload. Cardiac contractility remained largely unchanged compared to the pre-episode period. Electrocardiographic monitoring identified arrhythmias primarily consisting of atrial premature beats, ventricular premature beats, and ventricular escape beats. Given these clinical findings, the rapid tapering of corticosteroid therapy is considered the most probable contributing factor to the observed episodes. While the temporal association with the rapid steroid reduction is compelling, the persistent severe myocardial injury and (or) myocardial fibrosis and electrical remodeling likely contributed to the electrically unstable myocardial substrate. Consequently, the glucocorticoid dosage was adjusted and the patient was successfully weaned off mechanical ventilation on July 5. Despite the patient being in a state of hemodynamic stability at that moment, she had a documented history of recurrent episodes of ventricular fibrillation. This established a clear and high-risk substrate for sudden cardiac death. Following a formal cardiology consultation, it was collectively determined that the patient had a strong indication for secondary prevention of malignant arrhythmias. The implantable cardioverter-defibrillator (ICD) was therefore implanted as a necessary, prophylactic measure, and the patient was transferred to the Cardiology ICU for ICD placement on July 9. Meanwhile, coronary angiography revealed no significant coronary artery disease (**Video 4**). Ultimately, the patient recovered well and was discharged on July 29, 2024. Six-month follow-up revealed a stable clinical status, normal cardiac emzymes and intact cardiac function.

## Literature review

**Figure 3:**
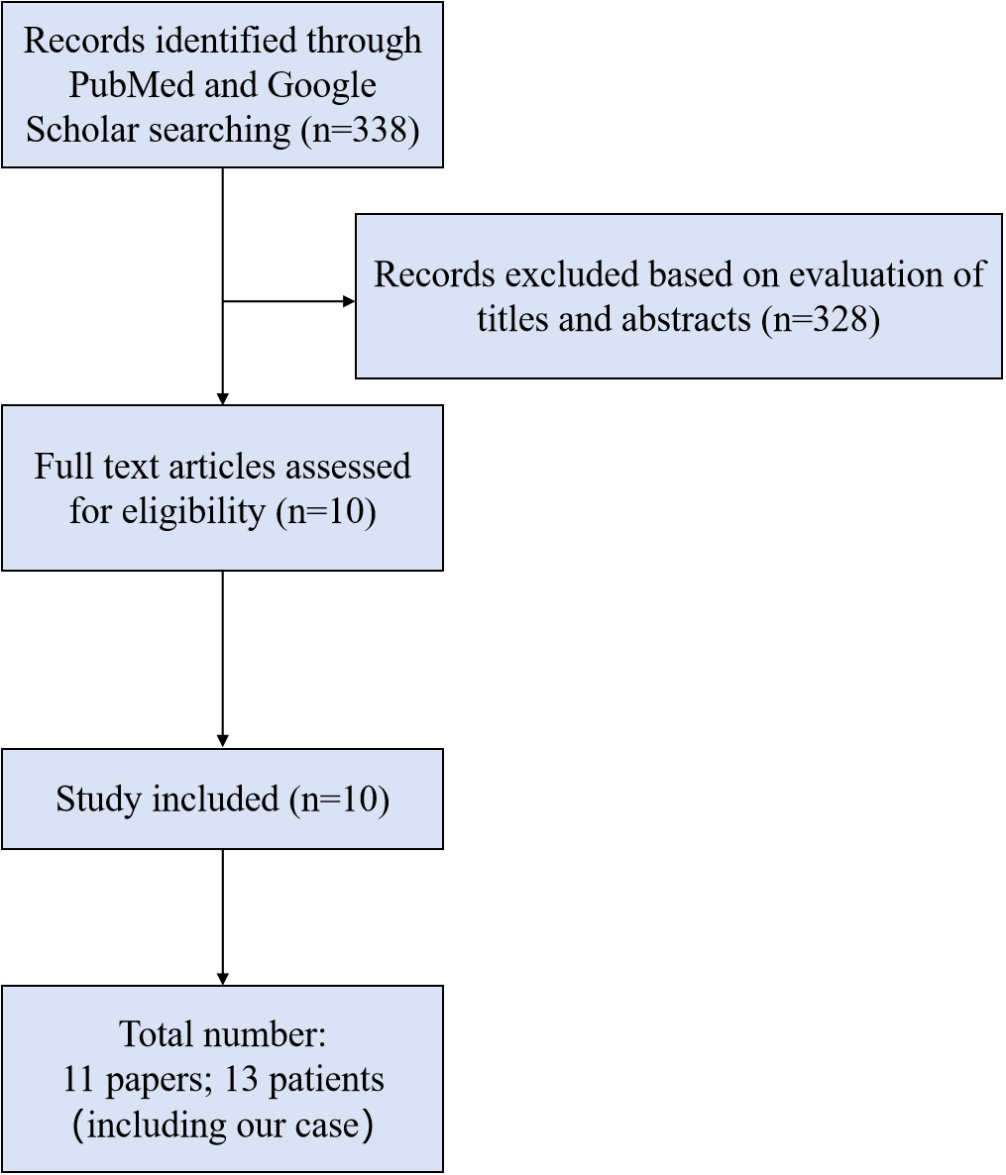
Screening process.

Through a comprehensive search of the PubMed and Google Scholar using various keywords such as “immune checkpoint inhibitor-induced myocarditis,” “immune checkpoint inhibitor-associated myocarditis,” “extracorporeal membrane oxygenation (ECMO),” and combinations thereof, we meticulously screened and excluded duplicate cases and irrelevant reports. Ultimately, 10 papers were identified, and a total of 12 patients were included in the analysis. **Figure 3** illustrates the screening process. **Supplementary Data Sheet 1** provides detailed characteristics of 13 patients with ICI-associated myocarditis who received ECMO across 11 included studies, including our present case.

Upon collecting relevant clinical data (**Table 1**), we observed that among the 13 patients, 6 (46.15%) were male and 7 (53.85%) were female. The mean patient age was 59.08 years, with males averaging 64.50 years and females 54.43 years. The types of malignancies included lung cancer (5 cases, 38.46%), urinary tract tumors (3 cases, 23.08%), melanoma (2 cases, 15.38%), gastrointestinal tumors (2 cases, 15.38%), and cervical cancer (1 case, 7.69%).

**Table 1:** Characteristics of patients treated with ECMO.

Characteristics	N = 13
Gender, no. (%)	
Male	6 (46.15)
Female	7 (53.85)
Age, mean±SEM, years	59.08 ± 3.60
Male	64.50 ± 2.35
Female	54.43 ± 6.04
Cancer type, no. (%)	
Lung cancer	5 (38.46)
Urinary tract tumor	3 (23.08)
Melanoma	2 (15.38)
Gastrointestinal tumor	2 (15.38)
Cervical cancer	1 (7.69)
ICI therapy, no. (%)	
Anti-PD-1	8 (61.54)
Anti-PD-1+Anti-CTLA-4	2 (15.38)
Anti-PD-L1	3 (23.08)
ICI treatment cycle, median (IQR), times	3.0 (2.0 ~ 7.0)
First administration to myocarditis, median (IQR), days	77.0 (20.5 ~ 250.0)
Duration of myocarditis, median (IQR), days	19.0 (15.5 ~ 42.5)
Laboratory data on admission, no. (%)	
Myocardial enzyme elevation	12 (92.31)
BNP (or NT-proBNP) elevation	8 (61.54)
Arrythmia, no. (%)	10 (76.92)
LVEF on admission, mean±SEM, %	38.31 ± 5.72
Diagnostic method, no. (%)	
Myocardial biopsy	8 (61.54)
Cardiac magnetic resonance imaging	2 (15.38)
Clinical diagnosis	3 (23.08)
Immunosuppressive therapy, No. (%)	
mPSL pulse therapy	9 (69.23)
Tacrolimus therapy	2 (15.38)
Mycophenolate mofetil	1 (7.69)
Intervention except for immunosuppression, No. (%)	
IVIg	8 (61.54)
PE	4 (30.77)
Infliximab	1 (7.69)
Left ventricular assist device, No. (%)	
ECMO	13 (100)
IABP	10 (76.92)
Impella CP	4 (30.77)
Duration of ECMO, median (IQR), days	9.0 (6.5 ~ 15.5)
Duration of IABP, median (IQR), days	11.5 (7.5 ~ 13.0)
Survived, no. (%)	9 (69.23)

ICI, Immune Checkpoint Inhibitor; ECMO, extracorporeal membrane oxygenation; IABP, intra-aortic balloon pump; LVEF, left ventricular ejection fraction; BNP, B-type natriuretic peptide; NT-proBNP, N-terminal pro-B-type natriuretic peptide; PD-1, programmed death-1; PD-L1, Programmed cell death ligand 1; CTLA-4, Cytotoxic T Lymphocyte-Associated Antigen 4; mPSL, methylprednisolone; PSL, prednisolone; IVIG, intravenous immunoglobulin; PE, plasma exchange; SME, standard error of mean; IQR, interquartile range; no., number.

Regarding ICI therapies, anti-PD-1 inhibitors were the most frequently administered (61.54%), followed by a combination of anti-PD-1 and anti-CTLA-4 inhibitors (15.38%) and anti-PD-L1 inhibitors (23.08%). Notably, monotherapy with nivolumab or pembrolizumab constituted the predominant ICI treatment. The median of ICI treatment cycle was 3.0 times (interquartile range (IQR), 2.0 to 7.0). Additionally, the median duration from first administration to myocarditis onset was 77.0 days (IQR, 20.5 to 250.0), while the mediam duration of myocarditis was 19.0 days (IQR, 15.5 to 42.5).

Clinically, in addition to nonspecific symptoms such as fever and dyspnea, over 90% of patients exhibited elevated myocardial enzymes at disease onset, while over 60% had elevated BNP or NT-proBNP levels. Furthermore, 76.92% of patients experienced arrhythmias, with an average left ventricular EF of 38.31% upon admission. Myocardial biopsy was the primary diagnostic method. Among the 13 patients, 10 underwent myocardial biopsy, 2 were diagnosed using cardiac magnetic resonance imaging (CMRI), and the remaining 3 were diagnosed clinically by excluding other conditions.

For the treatment of ICI-associated myocarditis, our findings indicate that 9 patients received high-dose corticosteroid therapy, 2 were treated with Tacrolimus, and 1 received Mycophenolate mofetil. In addition to immunosuppressive therapy, 10 out of the 13 ECMO-supported patients also received IABP therapy, while 4 had Impella CP placement. The median duration of ECMO and IABP support was 9.0 days (IQR, 6.5 to 15.5) and 11.5 days (IQR, 7.5 to 13.0), respectively. Ultimately, 9 of the 13 patients (69.23%) survived.

## Discussion

Our case report and literature review explore the application of ECMO in the management of ICI-associated myocarditis, a relatively rare but serious adverse event observed in cancer immunotherapy (6, 11). The incidence of ICI-associated myocarditis has increased in recent years with the expanded use of immune checkpoint inhibitors such as pembrolizumab, nivolumab, and ipilimumab, particularly in the treatment of various malignancies (3). Myocarditis, while infrequent, can present with life-threatening outcomes, including arrhythmias, heart failure, and even sudden cardiac arrest (3). Given its potential for rapid deterioration, early recognition and aggressive management are crucial.

In our case, a 41-year-old female with anal squamous cell carcinoma, developed fulminant myocarditis after receiving three cycles of pucotenlimab (anti-PD-1 therapy). Despite initial corticosteroid therapy for elevated troponin levels, she experienced sudden cardiac arrest, ventricular arrhythmias, and hemodynamic instability, necessitating VA-ECMO and IABP support. ECMO served as a bridge to provide temporary circulatory and respiratory support, allowing time for the myocardium to recover or for other therapeutic measures to take effect. A key conclusion from this case is the critical need for heightened clinical awareness and timely intervention in patients receiving ICIs, particularly those presenting with nonspecific symptoms such as fatigue, dyspnea, or chest pain, which may indicate of myocarditis.

ICI-associated myocarditis is a diagnosis of exclusion, primarily established based on the following key criteria, which are consistent with current international consensus guidelines (11, 12). First, the onset of clinical symptoms and cardiac biomarker elevation occurred shortly after initiation of ICI therapy, establishing a strong temporal association highly suggestive of an irAE. Second, other common causes of acute myocardial injury and cardiogenic shock, most notably acute coronary syndrome, were rigorously excluded. This was confirmed by coronary angiography, which showed no evidence of obstructive coronary artery disease. Third, the patient presented with diffuse, non-ischemic ST-T wave abnormalities and arrhythmias, consistent with an inflammatory myocardial process. Echocardiogram demonstrated global hypokinesia with severely reduced left ventricular systolic function. This pattern of global, rather than regional, wall motion abnormality is more characteristic of inflammatory myocarditis than of an ischemic insult. Concurrently, marked elevations in hs-TnI and NT-proBNP, confirming myocardial damage and hemodynamic stress. Furthermore, the co-occurrence of another irAE—specifically myositis—substantially increased the clinical probability of ICI-associated myocarditis, as multiple irAEs are known to manifest simultaneously. We acknowledge that endomyocardial biopsy (the histopathological gold standard) or CMRI could have provided further confirmation. However, given the patient’s life-threatening cardiogenic shock necessitating emergent VA-ECMO support, neither CMR nor invasive biopsy could be safely performed due to hemodynamic instability. Thus, the diagnosis was established clinically based on the above highly suggestive findings.

Studies showed that early initiation of ECMO has the potential to reduce mortality by providing critical support in the early phase of the disease (13, 14). In our review, ECMO was utilized in all cases with refractory shock, achieving a 69.23% survival rate, which is higher than previously reported (6). Although the number of reported cases remains relatively limited, there is growing evidence supports the use of ECMO in cases of refractory heart failure and shock (15). Several case series have demonstrated improved short-term survival outcomes, particularly when ECMO is used in conjunction with immunosuppressive therapies (16–18). However, long-term outcomes remain uncertain, with some patients experiencing ongoing cardiac dysfunction even after ECMO support is withdrawn (19, 20). More data from larger, multicenter studies are needed to better define the optimal indications, timing, and duration of ECMO support in this patient population.

The mechanism of ICI-associated myocarditis remains poorly understood but is thought to be driven by the disruption of immune tolerance, leading to the activation of autoreactive T cells targeting myocardial tissue (21). This immune-mediated damage, combined with the fact that patients receiving ICIs are often immunocompromised due to cancer treatment, presents a unique challenge in management. While corticosteroids and other immunosuppressive agents are the mainstay of treatment, their efficacy can be limited in severe cases, as highlighted in our case report and literature review. Therefore, ECMO may play a crucial role in the management algorithm for refractory cases, although it should be used cautiously given its associated risks, including bleeding, infection, and organ failure.

## Conclusion

Our analysis demonstrates ECMO’s potential as a bridge-to-recovery strategy for severe ICI-associated myocarditis with cardiogenic shock. The observed survival rate of 69.23% supports its judicious use in conjunction with prompt immunosuppression. Prospective studies are warranted to optimize ECMO initiation criteria, duration, and combination strategies with other circulatory support modalities.

## Data Availability

The original contributions presented in the study are included in the article/**Supplementary Material**. Further inquiries can be directed to the corresponding author.
