# Supplementary material for: Successful ECMO support for cardiogenic shock induced by immune checkpoint inhibitor-associated myocarditis: a case report and literature review
Source: Front Immunol. 2025 Dec 8;16:1646040. doi: 10.3389/fimmu.2025.1646040 (PMC12719444; doi:10.3389/fimmu.2025.1646040)
Supplement: Supplementary file 5 [file DataSheet1.docx]

**Supplementary Data 1.** A summary of cases of ECMO use in immune checkpoint inhibitor-associated myocarditis.

| Ref. | Age (year)/ sex | Medical history | Malignancy | ICI therapy | Cumulative number of cycles | Cumulative dose (mg) | First administration to myocarditis (days) | Duration of myocarditis (days) | Clinical symptoms | LVEF (on admission) | Lab tests (on admission) | Coronary angiography | Myocardial biopsy | Treatments | Outcome |
| --- | --- | --- | --- | --- | --- | --- | --- | --- | --- | --- | --- | --- | --- | --- | --- |
| Arangalage et al. (2017) | 35/F | Not particular | Melanoma | Ipilimumab(3 mg/kg), Nivolumab (1 mg/kg) | 1 | NA | 15 | 18 | Dyspnea, hyperthyroidism, heart failure, cardiogenic shock, arrythmia (sinus tachycardia, right bundle branch block, refractory ventricular tachycardia) | 50% | TnI 210 μg/L (normal < 0.045 μg/L), CK 11256 U/L (normal 24 ~ 192 U/L) | NA | No (CMRI confirmed) | ECMO (14 days)  mPSL (1 g/day)  IVIG (for 3 days)  PE  Tacrolimus | Survived |
| Frigeri et al. (2018) | 76/F | Not particular | Metastatic lung adenocarcinoma | Nivolumab (dose NA) | 7 | NA | 84 | 19 | Dyspnea, heart failure, cardiogenic shock | 15% | NT-proBNP 32447 ng/L; hs-cTnI 2674 ng/L | Normal | No (CMRI confirmed) | ECMO (12 days)  IABP (12 days)  mPSL (5 mg/kg/day)  PE  Infiximab (5 mg/kg, 3 doses) | Survived |
| Yamaguchi et al. (2018) | 60/M | Not particular | Melanoma | Nivolumab (2 mg/kg) | 13 | NA | 365 | 34 | Fatigue, fever, hypotension, arrhythmia | 70% | NA | Normal | Yes | ECMO (8 days)  IABP (9 days)  PSL (1000 mg/day for 3 days)  IVIG (50 g/day for 2 days) | Survived |
| Imai et al. (2018) | 70/M | Hypertension | Lung squamous cell carcinoma | Pembrolizumab (200 mg/body) | 2 | 400 | 35 | 49 | Fever, faintness, muscle weakness, hypotension, cardiogenic shock | 29% | TnT 17.6 ng/mL (normal <0.014 ng/mL), CK 9786 U/L (normal 57 ~ 218 U/L), CK-MB 154 U/L (normal <30 U/L) | Normal | Yes | ECMO (7 days)  IABP (13 days)  mPSL (1 g/day for 3 days), PSL (10 mg)  IVIG (1 g/kg for 2 days)  Tacrolimus | Died |
| Wang et al. (2021) | 56/M | NA | Colonic carcinoma | Nivolumab (140 mg daily) | 6 | 840 | 135 | 14 | Progressive chest tightness and pain, cardiogenic shock, arrythmia (sinus bradycardia, arrhythmia, wide QRS), pneumoniae | 50% | TnT 0.23 ng/mL (normal < 0.014 ng/mL), CK-MB 8.99 ng/mL (normal <4.87 ng/mL), NT-proBNP 180.40 ng/L (normal <125 ng/L) | Normal | NA | ECMO (8 days)  IABP (NA)  mPSL (160 mg/day for 3 days, followed by 80 mg/ day)  IVIG (25 /day for 5 days) | Died |
| Matsui et al. (2020) | 69/M | Not particular | Metastatic upper urinary tract urothelial carcinoma | Pembrolizumab  (200 mg/body) | 2 | 400 | 26 | 17 | Myalgia, fatigue, muscle weakness, cardiac arrest, cardiogenic shock, arrythmia (complex ventricular rhythm, complete atrioventricular block, wide QRS) | 50% | hs-cTnI 10318 pg/mL, CK 3887 U/L, CK-Mb 143 U/L, Myo 3800 ng/mL | Normal | Yes | ECMO (17 days)  IABP (17 days)  mPSL (1000 mg/day, for 3 days), PSL (1 mg/kg/day)  PE | Died |
| Izumi et al. (2024) | 69/M | Hypertension | Prostate adenocarcinoma | Pembrolizumab (200 mg/body) | 7 | 1400 | 1020 | NA | Fever, chest pain, decompensated heart failure, cardiogenic shock, arrythmia (complete atrioventricular block, ventricular tachycardia, paroxysmal atrial fbrillation, wide QRS) | 17% | TnT 48.12 ng/mL, CK 2450 U/L, CK-MB 365 U/L; BNP 921.7 pg/mL; CRP 9.32 mg/dL | Normal | Yes | ECMO (6 days)  IABP (13 days)  Impella CP  mPSL (1000 mg, 3 days), PSL (10 mg) | Died |
|  | 63/M | Not particular | Poorly differentiated lung cancer | Atezolizumab (1200 mg) | 31 | NA | 692 | NA | Fever, chest pain, decompensated heart failure, cardiogenic shock, arrythmia (complete atrioventricular block, wide QRS complex) | 10% | TnT 11ng/mL, CK 3859 U/L, CK-MB 61 U/L; BNP 388.9 pg/mL, CRP 9.47 mg/dL | Normal | Yes | ECMO (13 days)  IABP (3 days)  Impella CP  mPSL (1000 mg, 3 days) | Died |
|  | 76/F | Hypertension, ischemic heart disease | Lung adenocarcinoma | Atezolizumab (1200 mg) | 1 | NA | 11 | NA | Fatigue, dyspnea, decompensated heart failure, cardiogenic shock, arrythmia (ventricular tachycardia, paroxysmal atrial fbrillation, normal QRS width) | 10% | TnT 0.23ng/mL, CK 101 U/L, CK-MB 5 U/L, BNP 1357.3 pg/mL, CRP 0.59 mg/dL | Normal | Yes | ECMO (17 days)  Impella CP  mPSL (1000 mg, 3 days), PSL (1 mg/kg) | Survived |
| Wang et al. (2020) | 50/F | Not particular | Nonsmall cell lung cancer | Toripalimab (dose NA) | 2 | NA | 15 | NA | Palpitation, dyspnea, cardiogenic shock, arrythmia (ventricular premature contractions, ventricular tachycardia, atrial fibrillation, third-degree atrioventricular block) | 52% | TnT 1.87 μg/L (normal 0 ~ 0.05 μg/L), CK-MB 285 U/L (normal 1 ~ 18 U/L), CK 4800 U/L (normal 55 ~ 170 U/L), BNP 85.8 pg/ mL (normal 5 ~ 100 pg/mL) | Normal | No | ECMO (6 days)  IABP (7 days)  mPSL (120 mg/d)  IVIG (20 g/d) | Survived |
| Ramayya et al. (2022) | 50/F | Not particular | Metastatic cervical cancer | Atezolizumab (dose NA) | 2 | NA | 42 | 36 | Dyspnea, chest pain, lower extremity edema, diplopia, heart failure, hypotensive, arrythmia (inferior Q waves, ventricular arrhythmias) | 60% | TnI 1.4 ng/mL (normal < 0.32 ng/mL), BNP 11 pg/mL (normal 10 ~ 100 pg/mL) | Normal | Yes | ECMO (18 days)  mPSL (1000 mg, for 3 days), PSL (60 mg daily)  IVIG (400 mg/kg)  Abatacept (11.4 mg/kg) | Survived |
| Itzhaki et al. (2019) | 53/F | Smoking | Metastatic renal cell carcinoma | Ipilimumab (dose NA);  Nivolumab (dose NA) | 3 | NA | 77 | 14 | Dizziness, cardiogenic shock  arrythmia (premature ventricular beats, ventricular tachycardia) | 30% | hs-cTnT 2469 ng/L (normal 0 - 14 ng/L); NT-proBNP 8840 pg/mL (normal adjusted for age and sex, <249 pg/mL) | Normal | Yes | ECMO (3 days)  IABP (NA)  mPSL (1000 mg, for 3 days), PSL (1 mg/kg/day)  IVIG (total dose of 330 mg divided over 5 daily doses)  Mycophenolate mofetil | Survived |
| Our case | 41/F | Not particular | Anal canal squamous cell carcinoma | Pucotenlimab (200 mg/body) | 3 | 600 | 126 | 66 | Chest tightness，lower limb pain, fatigue, dizziness, cardiogenic shock, arrythmia (sinus tachycardia, atrial fibrillation, paroxysmal polymorphic ventricular tachycardia, torsades de pointes, complete right bundle branch block, Q-T interval prolongation) | 55% | hs-cTnI 16106.1 ng/L, CK 522U/L，CK-MB 51.5 ng/mL，BNP 587.5 pg/mL | Normal | No | ECMO (9days)  IABP (11 days)  Impella CP  mPSL (1000 mg, for 3 days)  PE (2000 for 5 times)  IVIG (1g/day) | Survived |

M, male; F, female; ICI, Immune Checkpoint Inhibitor; ECMO, extracorporeal membrane oxygenation; IABP, intra-aortic balloon pump; LVEF, left ventricular ejection fraction; CMRI, cardiac magnetic resonance imaging; hs-cTnI, high-sensitive cardiac troponin I; TnI, troponin I; TnT, troponin T; CK, creatine kinase ; CK-MB, CK-myocardial band; Myo, myoglobin; BNP, B-type natriuretic peptide; NT-proBNP, N-terminal pro-B-type natriuretic peptide; CRP, C-reactive protein; mPSL, methylprednisolone; PSL, prednisolone; IVIG, intravenous immunoglobulin; PE, plasma exchange; NA, not available.
